# The Trade-Off between Spatial and Temporal Variabilities in Reciprocal Upper-Limb Aiming Movements of Different Durations

**DOI:** 10.1371/journal.pone.0097447

**Published:** 2014-05-16

**Authors:** Frederic Danion, Raoul M. Bongers, Reinoud J. Bootsma

**Affiliations:** 1 Aix Marseille Université, CNRS, Institut de Neurosciences de la Timone UMR 7289, Marseille, France; 2 University of Groningen, University Medical Center Groningen, Center for Human Science, Groningen, The Netherlands; 3 Aix Marseille Université, CNRS, Institut des Sciences du Mouvement UMR 7287, Marseille, France; VU University Amsterdam, The Netherlands

## Abstract

The spatial and temporal aspects of movement variability have typically been studied separately. As a result the relationship between spatial and temporal variabilities remains largely unknown. In two experiments we examined the evolution and covariation of spatial and temporal variabilities over variations in the duration of reciprocal aiming movements. Experiments differed in settings: In Experiment 1 participants moved unperturbed whereas in Experiment 2 they were confronted with an elastic force field. Different movement durations—for a constant inter-target distance—were either evoked by imposing spatial accuracy constraints while requiring participants to move as fast as possible, or prescribed by means of an auditory metronome while requiring participants to maximize spatial accuracy. Analyses focused on absolute and relative variabilities, respectively captured by the standard deviation (SD) and the coefficient of variation (CV = SD/mean). Spatial variability (both SDspace and CVspace) decreased with movement duration, while temporal variability (both SDtime and CVtime) increased with movement duration. We found strong negative correlations between spatial and temporal variabilities over variations in movement duration, whether the variability examined was absolute or relative. These findings observed at the level of the full movement contrasted with the findings observed at the level of the separate acceleration and deceleration phases of movement. During the separate acceleration and deceleration phases both spatial and temporal variabilities (SD and CV) were found to increase with their respective durations, leading to positive correlations between them. Moreover, variability was generally larger at the level of the constituent movement phases than at the level of the full movement. The general pattern of results was robust, as it emerged in both tasks in each of the two experiments. We conclude that feedback mechanisms operating to maximize task performance are subjected to a form of competition between spatial and temporal variabilities.

## Introduction

The ability to accurately control our movements in space and time is essential for every-day behavior. Yet, all our movements are intrinsically variable and we (can) never repeat the same movement twice. Understanding the origins of variability and the processes involved in controlling its influence on performance are central themes in the fields of motor control and computational neuroscience [1]–[11]. However, when addressing the spatial and the temporal aspects of the variability of movement, empirical as well as theoretical work has essentially advanced along different lines. In calling upon dedicated experimental paradigms the large majority of studies has focused exclusively on either the spatial or the temporal aspects of movement variability. Moreover, the variability measures studied have most often reflected absolute variability, expressed in the same dimensional units as the mean; the standard deviation (SD) of movement parameters (such as duration or distance covered) is the most widely used. If absolute variability is proportional to the mean, thus following Weber's law, relative variability is constant. Yet, while clearly informative, relative variability—as captured by the coefficient of variation CV, defined by the ratio of SD over the mean—has been much less widely used. As a result, joint analyses of spatial and temporal variabilities are few and far between (but see [12] for an early discussion). In the present contribution we examined how (absolute and relative) spatial and temporal variabilities co-evolve over variations in the duration of reciprocal upper-limb aiming movements.

The relation between the duration of movement and *temporal variability* has been addressed in several different tasks. Following up on the work of Wing and Kristofferson [13], a first experimental paradigm relies on finger-tapping tasks, in which participants are asked to synchronize their tapping movements with the beeps of a metronome. When varying the rate of tapping (and incidentally the duration of the tapping movement), the SD of the inter-tap interval increases with the length of the tapping period [13]–[16]. The picture is less clear for the relative temporal variability of tapping movements [14], [17]. Several studies reported that CV decreased with the duration of the inter-tap interval [14], [15], [18], [19]; occasionally the opposite pattern has also been found [20]. However, because of the focus on timing properties, movement amplitude is rarely controlled or even measured in this kind of task [19], [21]).

This confounding factor is controlled in two other paradigms used for studying the effect of movement duration on temporal variability. Movement duration is varied either directly by requiring participants to move through a designated distance while matching a prescribed movement time (MT) or indirectly by requiring participants to move a fixed distance so as to intercept a target moving along a path perpendicular to the direction of participant's hand motion. In the latter case, different MTs can be evoked by varying the speed of the moving target and, to a lesser degree, the time window available for interception (i.e., target size). In both these discrete movement paradigms absolute temporal variability (i.e. SD of MT) has been found to increase with increasing MT [22]–[28], but to our knowledge the relation between MT and the CV of MT has remained largely unexplored in these tasks. We are aware of reports in which CV of MT was manipulated but changes in MT were obtained through changes in the amplitude of movement [29]. Although sometimes not explicitly computed, further examinations of studies reporting means and SDs suggest that CV of MT may (slightly) increase with MT [3], [30]. Overall the state of affairs speaks for a more detailed analysis of MT variability.

The relation between movement duration and *spatial variability* has essentially been studied in the framework of two dedicated experimental paradigms. In the first, which we will refer to as a Fitts task, participants have to reach a target as rapidly as possible. Movement distance (MD) and absolute spatial variability are controlled by manipulating, respectively, the location and the width of the target to be reached [2], [31]. The participants' task is to minimize MT. In the second task, that we will refer to as a Schmidt task [3], [29], participants have to move to a target line within a prescribed MT. Here, final spatial deviation from the target line is to be minimized. In the Fitts task, variations in MT are evoked by variations in task difficulty (defined by the ratio of distance to be covered and the spatial tolerance provided by the target width). In the Schmidt task, MT is prescribed. The discrete and reciprocal versions of both paradigms have proven highly valuable in demonstrating speed-accuracy trade-offs in aiming movements, revealing that, for a given movement amplitude, shorter MTs are associated with larger absolute spatial variability [3], [4], [32]. However, due to the focus on spatial aspects of the task, temporal variability has typically not been an object of interest in these paradigms.

Overall, it appears that the influence of movement duration on spatial variability and on temporal variability has generally been studied separately, using a variety of experimental paradigms and motor tasks. As a result, it remains unclear whether conclusions on timing variability derived in movement timing, target interception or finger-tapping tasks also hold in a task where MT is emergent (as in the Fitts task) and a task in which MT is prescribed (as in the Schmidt task). Additionally, the fact that temporal and spatial aspects of movement variability have typically been studied separately makes that the relation between these two types of variability remains largely unknown. Finally, it is not known whether this relation is generic or dependent on the specifics of the task and its setting, and whether this relation is similar for absolute and relative variabilities.

In the present study we manipulated MT during reciprocal aiming in two different experimental tasks (the Fitts task evoking MT and the Schmidt task imposing MT) in order to examine whether and if so, how, spatial variability and temporal variability vary with MT. Moreover, we sought to determine how spatial and temporal variabilities relate to one another, within a given task and setting and across tasks and settings. To this end, we analyzed how movement duration affected spatial variability and temporal variability in the Fitts and Schmidt tasks in two different settings. In Experiment 1, participants performed both reciprocal aiming tasks by moving a hand-held stylus horizontally across the surface of a graphics tablet. We refer to this setting as the standard setting. In Experiment 2, participants performed the same two reciprocal aiming tasks by horizontally displacing a hand-held object in the presence of an elastic force-field. We refer to this setting as the elastic force-field setting. These different settings allowed testing the generality of our findings.

What might we expect for the relation between spatial and temporal variabilities when MT is varied? Concerning absolute variability we can make a straightforward prediction. Since SD of MT is expected to increase with MT [3], whereas SD of MD is expected to decrease with MT [2], we predict a negative correlation between spatial and temporal SDs. In contrast, predicting the relation between relative spatial and temporal variabilities is more tricky because it is unclear how the CV of MT will change as a function of MT. Using a hybrid reciprocal aiming task, combining spatial constraints from the Fitts task (fixed-size targets) and temporal constraints from the Schmidt task (metronome matching), Shafir and Brown [33] reported effects of variations in MT on both relative spatial variability and relative temporal variability for single-joint movements. Relative spatial variability decreased with increasing movement duration, while relative timing variability increased for the longest (500 ms) studied MT only. For multi-joint movements, as studied in the present contribution, they did not find any effects of MT on either relative spatial or relative temporal variability at the level of the hand (end effector). Thus from the existing literature it is not clear what to expect in terms of relative variabilities. We reasoned that if relative temporal and spatial variabilities are driven by common neural processes, they should increase or decrease in concert [34], [35]. Alternatively, one could envisage that minimizing temporal and minimizing spatial variability are driven by separate processes competing for the same resources. In that case, minimizing relative spatial variability would be obtained at the expense of higher relative temporal variability, and vice-versa.

Finally, following up on Woodworth's (1899) two-component model of limb control, it has been advocated that the acceleration phase of an aiming movement (up to peak velocity) is controlled in a feedforward manner, whereas its deceleration phase (from peak velocity onward) is controlled in a feedback manner [36], [37]. A second objective of our study was to investigate spatial and temporal variabilities within each of these two phases of the movement as well as the relation between the two. The rationale was to isolate the possible contribution of feedforward and feedback mechanisms in the minimization of space and time variabilities, as well as in the relation between space and time variabilities.

## Methods

### Ethics statement

For Experiment 1 we reanalyzed a part of the raw dataset of a previously published study [38]. These data were collected at the ISM in Marseille in 2006. Both experiments reported here were approved by the local institutional review board (*Comité d'Ethique de l'Institut des Sciences du Mouvement d'Aix-Marseille Université*). In both cases, written informed consent was obtained prior to the study. Both studies were performed in accordance with local University regulations and the 1964 Declaration of Helsinki. None of the participants received compensation. None of the participants were younger than 18.

### Participants

Two different groups of ten participants (5 males and 5 females) participated in each experiment (mean age ±SD; Exp. 1: 24.5±7.3 years; Exp. 2: 23.8±5.8 years). All participants were right-handed and had normal or corrected-to-normal vision.

### Task and apparatus

In both experiments participants performed two reciprocal aiming tasks. In the (evoked MT) Fitts task they were to move as fast as possible between two target bars whose widths were varied over experimental conditions. Different MTs were evoked by manipulating the spatial precision requirements defining task difficulty. In the (prescribed MT) Schmidt task they were to move at the rhythm of an auditory metronome between two lines while minimizing spatial deviation from the target lines. MT was varied over experimental conditions by manipulating the metronome frequency. The two experiments differed in their settings: Experiment 1 was performed in a standard reciprocal aiming setting and Experiment 2 in a setting with an elastic force-field.

In Experiment 1 we report the results of a re-analysis of the raw data collected for the study of Bongers et al [38]. Two experiments were actually reported in [38]. For convenience purposes the current study focused on the (second) experiment in which movement durations were kept similar for the different conditions of the two aiming tasks (the same results were obtained when reanalyzing both experiments). In this study the Schmidt and Fitts tasks were performed by sliding a hand-held non-marking stylus horizontally between two vertically elongated target bars (Fitts task) or between two vertical line segments (Schmidt task) depicted on a A3-sized sheet of paper placed in the landscape orientation on top of a Wacom Ultrapad A3 graphics tablet. The tablet was placed on the tabletop in front of the seated participant, such that hand movement was in the left-right direction. Stylus motion was sampled at 170 Hz. Further technical details and procedures related to Experiment 1 can be in found in our original paper [38].

In Experiment 2 seated participants performed the same two tasks with a 0.045-kg object held between the thumb and the index finger of the right hand at a height of about 20 cm above the tabletop. The left side of the object was attached to an elastic cord (stiffness 25 N/m) fixed to a vertical bar firmly attached to the left side of the table (for rather similar setups see [39]–[41]. This setup gave rise to an elastic load force (LF) that varied as a linear function of hand position (*r*>0.99). LF was measured with a force sensor (ELPM-T1M-25N, Entran, Fairfield, NJ, USA) attached to the elastic cord at its world fixation. LF, sampled at 1000 Hz, determined the horizontal position of a white rectangular cursor (4 mm high by 2 mm wide) depicted on a 20-inch LCD monitor (1600×1200 pixel resolution; 75 Hz refresh rate) placed at a distance of 60 cm in front of the participant. Their task was to move the screen cursor between two vertically elongated target bars (Fitts task) or between two vertical line segments (Schmidt task) depicted on the screen.

### Procedure

In Experiment 1, all participants started with the (evoked MT) Fitts task. For each of four possible inter-target distances (D = 5, 10, 20, or 30 cm), six level of task difficulty (ID =  log_2_(2D/W)  = 3.5, 4.0, 4.5, 5.0, 5.5, or 6.0) were created by adapting target width W. In the (prescribed MT) Schmidt task, the experimental design was set with 6 movement durations adjusted for each participant to match his/her mean movement duration in the evoked MT task (MTs ranging from 371 to 902 ms) in each of the same four inter-target distances. For each task, each participant performed one trial under each experimental condition representing a total of 48 trials (6×4×2). Trial duration was adjusted so as to result in an average of about 60 aiming movements (i.e., half cycles).

In Experiment 2, half of the participants started with the (evoked MT) Fitts task, followed by the (prescribed MT) Schmidt task, while this order was reversed for the other half. The center of the left target corresponded to an elastic LF on the object of 3 N and the center of the right target corresponded to an elastic LF of 7 N, for an inter-target distance of 16 cm. In the (evoked MT) Fitts task, the width of the targets was varied across experimental conditions so as to obtain ID = 3.0, 4.0, 5.0, or 6.0. In the (prescribed MT) Schmidt task, the experimental design was set with 4 movement durations (MTs ranging from 323 to 1000 ms) whose values were selected based on pilot data, so as to approximate the MTs associated with each ID during the (evoked MT) Fitts task. In both sessions, each participant performed 4 trials of 25 s in each experimental condition (ID or MT), representing a total of 32 trials (4×4×2). Each session was preceded by 2 or 3 training trials to familiarize the participant with the task.

Task instructions were similar in the two experiments. In the Fitts task participants were instructed to move as fast as possible between the two target bars while reversing movement direction within the target area. In the Schmidt task participants were instructed to move between the target lines at the rhythm prescribed by the auditory metronome while reversing movement direction as close as possible to the target lines.

### Data analysis

In Experiment 1, half cycles 11 to 50 (i.e., 40 movements between targets) were used for the analyses whereas in Experiment 2 all half cycles performed during the last 20 s of each trial were analyzed. To determine movement distance (MD) and movement time (MT), the reversal points were detected for each half cycle from the extremes in the raw position data along the horizontal axis. From these reversal positions MD and MT were computed for each aiming movement. Subsequently we computed, for each trial, a mean and a standard deviation of MD and MT, as well as a temporal CV (CVtime  = SD of MT/mean MT) and a spatial CV (CVspace  = SD of MD/mean MD).

Spatial and temporal variabilities were also examined separately for the acceleration and deceleration phases of each aiming movement. The acceleration phase was defined as the portion of movement from movement onset (a reversal point) up to peak velocity. The deceleration phase was defined as the portion of movement from peak velocity to movement offset (next reversal point). For each phase of the movement, the mean, SD and CV of both duration and distance covered was computed over each trial.

The main statistical analyses used in this study were analyses of variance (ANOVA). We used ANOVAs with repeated-measures on the factor ID for the Fitts task and on the factor Prescribed MT for the Schmidt task. These factors had 6 levels in Experiment 1 and 4 levels in Experiment 2. Note that for the sake of simplicity and with respect to the goal of the study, the effect of inter-target distance, manipulated in Experiment 1, was not addressed (i.e., data were pooled over the 4 inter-target distances). If sphericity could not be assumed we used Greenhouse-Geisser corrections for the degrees of freedom. Whenever necessary the Newman-Keuls technique was used for post-hoc analyses. The relation between CVtime and CVspace was examined by correlation analyses using group averaged data as well as individual data. A 0.05 significance threshold was used for all analyses.

## Results

### Experiment 1: reciprocal aiming in the standard setting

The means, standard deviations and coefficients of variation of the durations and distances covered can be found in Table S1, for the full aiming movement and for each of the two movement phases considered separately.

#### Full movement

As predicted by Fitts' law and in line with our intentions, increases in task difficulty gave rise to systematic increases in MT. Correlation analysis of group means revealed a strong linear relation, *r*(4)  = 0.99, *p*<0.001, corroborated by a linear regression analysis: MT = −413+216×ID, *F*(1, 5)  = 299.74, *p*<0.001. Analyses for individual participants revealed similar results, with coefficients of correlation between MT and ID ranging from 0.97 to 0.99. Overall MT changed substantially across IDs, from an average of 371 ms under ID = 3.5 to 902 ms under ID = 6. In the Schmidt task participants almost perfectly reproduced the MTs prescribed by the metronome. Averaged across all experimental conditions, mean absolute error between MTs of all individual aiming movements and the prescribed MT was 0.3%.

In the (evoked MT) Fitts task both absolute and relative spatial variability decreased over ID, that is with increasing MT. This result was of course expected, as Fitts law stipulates that evoked MT is a logarithmic function of the task's relative spatial precision requirements. As expected absolute temporal variability increased with MT, but since this increase was faster than linear it also led to an increase in relative temporal variability with increasing MT (Table S1). A similar pattern of results was observed in the (prescribed MT) Schmidt task: both absolute and relative spatial variabilities decreased with increasing MT, while both absolute and relative temporal variabilities increased with increasing MT. These observations were corroborated by ANOVAs with repeated measures on the factors ID (Fitts task) or Prescribed MT (Schmidt task). The results of these statistical analyses are reported in Table 1.

**Table 1:** Analysis of Variance results for Experiment 1 (standard setting).

		Effect of ID in Fitts Task			Effect of MTp in Schmidt Task		
		*F*	*df*	*P*	*F*	*df*	*P*
***Full Movement***	SD time	65.98	5, 45	<0.001	49.58	1.4, 12.3	<0.001
	SD space	76.65	1.8, 16.0	<0.001	32.47	5, 45	<0.001
	CV time	7.32	2.7, 24.6	<0.001	7.16	2.2, 19.6	<0.01
	CV space	82.58	1.6, 14.2	<0.001	41.07	5, 45	<0.001
***Accel. Phase***	SD time	30.88	5, 45	<0.001	75.03	2.0, 17.7	<0.001
	SD space	2.77	5, 45	<0.05	20.25	1.9, 16.8	<0.001
	CV time	13.75	5, 45	<0.001	18.14	1.5, 13.7	<0.001
	CV space	15.72	5, 45	<0.001	21.65	1.7, 15.4	<0.001
***Decel. Phase***	SD time	85.49	5, 45	<0.001	85.27	2.0, 18.3	<0.001
	SD space	3.83	5, 45	<0.01	22.22	1.8, 16.4	<0.001
	CV time	1.71	5, 45	= 0.153	12.56	2.7, 17.7	<0.001
	CV space	1.76	5, 45	= 0.140	15.41	2.2, 20.1	<0.001

ID =  Index of Difficulty. MTp =  prescribed movement time.

#### Movement phases

In line with the literature [42]–[44], the lengthening of MT evoked by increasing ID in the Fitts task was characterized by a rising asymmetry in the durations of the acceleration (increasing from 187 to 339 ms) and deceleration (increasing from 184 to 563 ms) phases. As a result the percentage of total movement time spent accelerating decreased with ID from 50.5 to 37.6%.

A qualitatively similar pattern of results was observed in the (prescribed MT) Schmidt task, where the percentage of total movement time spent accelerating decreased from 53.9 to 45.7%. Here too the durations of the acceleration and deceleration phases increased asymmetrically with total MT (respectively, from 200 to 411 ms and from 171 to 489 ms). The effect of MT on the asymmetry was stronger in the (evoked MT) Fitts task than in the (prescribed MT) Schmidt task.

Contrary to what was observed for the full movement, for both the acceleration and the deceleration phase of the movement, the absolute variability of the distance covered in each subphase increased (rather than decreased) with MT; absolute temporal variability increased with MT, as was observed for the full movement. This pattern of results emerged in the (evoked MT) Fitts task as well as in the (prescribed MT) Schmidt task (Table S1). Moreover, in both tasks relative spatial variability and relative temporal variability increased with MT during the acceleration phase. During the deceleration phase, relative spatial and temporal variabilities increased with MT for the Schmidt task but not for the Fitts task. As shown in Table 1, these observations were corroborated by ANOVAs with repeated measures on the factor ID (Fitts task) or Prescribed MT (Schmidt task).

#### Covariation of spatial and temporal variabilities

**Figure 1:**
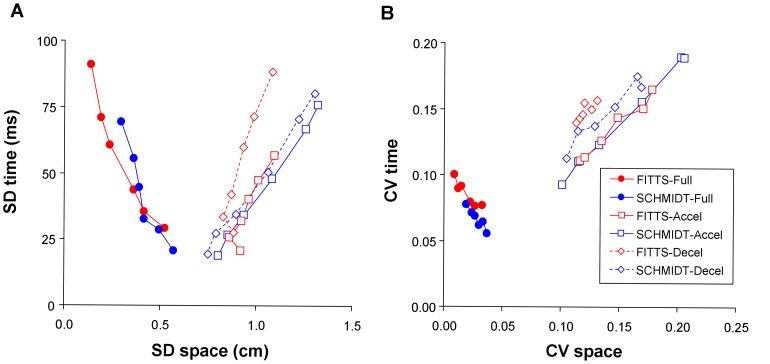
Relations between temporal and spatial variabilities observed in Experiment 1 (standard setting) over the full movement and the constituent acceleration and deceleration phases. Space-time relations for absolute variability (A) and relative variability (B).

Together the results of Experiment 1 demonstrated that, for both the Fitts and Schmidt tasks, a trade-off between spatial and temporal variabilities is observed at the level of the full movement, whether variability be expressed in absolute (Fig. 1A) or relative (Fig. 1B) terms. SDspace and SDtime were strongly negatively correlated (Fitts: *r*(4)  = −0.96, *p*<0.01; Schmidt: *r*(4)  = −0.95, *p*<0.01). Similar results were obtained for correlations between CVspace and CVtime (Fitts: *r*(4)  = −0.92, *p*<0.05; Schmidt: *r*(4)  = −0.96, *p*<0.01).

If MT-related variations in CVspace and CVtime were fully compensatory, their sum CVtotal  =  CVspace + CVtime would not vary with MT. CVtotal and MT were indeed not systematically related, as revealed by correlation coefficients that remained far from significance for both tasks (*p*'s>0.3). These observations were corroborated by one-way ANOVAs on CVtotal with repeated measures on the factor ID (Fitts task) or Prescribed MT (Schmidt task) showing no significant effects (*F*(5, 45) <1, *ns*). Overall our results indicate that when CVspace decreased by 1%, CVtime increased by 1% (and vice versa). In the Fitts task mean CVtotal was 10.5±0.4% (SD across IDs) and in the Schmidt task it was 9.5±0.2% (SD across prescribed MTs).

When movement phases were considered separately, spatial and temporal variabilities were found to covary positively both in absolute (Fig. 1A) and relative terms (Fig. 1B). Further examination of relative variability showed that for the acceleration phase the correlations between CVtime and CVspace were *r*(4)  = +0.98, *p*<0.001 (Fitts task), and +0.99 *p*<0.001 (Schmidt task). For the deceleration phase the correlations between CVtime and CVspace were *r*(4)  = +0.84, *p*<0.05 (Fitts), and *r*(4)  = +0.97, *p*<0.001(Schmidt task).

### Experiment 2: reciprocal aiming in an elastic force-field setting

The means, standard deviations and coefficients of variation of the durations and distances covered can be found in Table S2, for the full aiming movement and for each of the two movement phases considered separately.

#### Full movement

Results were very similar to those obtained in Experiment 1. Indeed, as predicted by Fitts' law, increases in ID once again gave rise to systematic increases in MT. Correlation analysis of group means revealed a strong linear relation *r*(2)  = 0.99, *p*<0.01, corroborated by a linear regression analysis: MT = −632+305×ID, *F*(3, 27)  = 270.92, *p*<0.001. Analyses for individual participants revealed similar results with *r* values ranging from 0.97 to 1.00 (mean  = 0.99). Overall MT changed substantially across IDs, from 334 ms under ID = 3 to 1241 ms under ID = 6. In the Schmidt task participants again almost perfectly reproduced the MTs prescribed by the metronome. Averaged across all experimental conditions, mean absolute error between MTs of all individual aiming movements and the prescribed MT was 4.7%.

In the (evoked MT) Fitts task both absolute and relative spatial variability decreased over ID, that is with increasing MT, as expected on the basis of Fitts law. Absolute temporal variability increased faster than linear with MT, leading to an increase in relative temporal variability with increasing MT (Table S2). Similar results were observed in the (prescribed MT) Schmidt task: both absolute and relative spatial variabilities decreased with increasing MT and both absolute and relative temporal variability increased with increasing MT. These observations were corroborated by ANOVAs with repeated measures on the factors ID (Fitts task) and Prescribed MT (Schmidt task). The results of these statistical analyses are reported in Table 2.

**Table 2:** Analysis of Variance results for Experiment 2 (elastic force-field setting).

		Effect of ID in Fitts Task			Effect of MTp in Schmidt Task		
		*F*	*df*	*P*	*F*	*df*	*P*
***Full Movement***	SD time	178.39	3, 27	<0.001	76.28	3, 27	<0.001
	SD space	146.60	3, 27	<0.001	39.61	3, 27	<0.001
	CV time	18.26	3, 27	<0.001	24.56	3, 27	<0.01
	CV space	142.00	3, 27	<0.001	46.19	3, 27	<0.001
***Accel. Phase***	SD time	33.37	3, 27	<0.001	84.79	3, 27	<0.001
	SD space	4.92	3, 27	<0.01	28.10	3, 27	<0.001
	CV time	7.63	3, 27	<0.01	27.48	3, 27	<0.001
	CV space	15.39	3, 27	<0.001	27.10	3, 27	<0.001
***Decel. Phase***	SD time	120.70	3, 27	<0.001	97.28	3, 27	<0.001
	SD space	4.07	3, 27	<0.05	12.69	3, 27	<0.001
	CV time	4.38	3, 27	<0.05	27.78	3, 27	<0.001
	CV space	5.29	3, 27	<0.01	9.22	3, 27	<0.001

ID =  Index of Difficulty. MTp =  prescribed movement time.

#### Movement phases

As in Experiment 1, the lengthening of MT evoked by increasing ID in the Fitts task was characterized by a rising asymmetry in the durations of the acceleration (increasing from 174 to 403 ms) and deceleration (increasing from 160 to 838 ms) phases. As a result the percentage of total movement time spent accelerating decreased with ID from 52.1 to 32.5%.

A qualitatively similar pattern of results was observed in the (prescribed MT) Schmidt task, where the percentage of total movement time spent accelerating decreased from 52.4 to 38.7%. Here too the durations of the acceleration and deceleration phases increased asymmetrically with total MT (respectively, from 169 to 386 ms and from 153 to 612 ms). Note again that the effect of MT on the asymmetry was not as strong in the (prescribed MT) Schmidt task as in the (evoked MT) Fitts task.

Contrary to what was observed for the full movement, for both the acceleration and the deceleration phases, SD space increased (rather than decreased) with MT in the Schmidt task. In the Fitts task SD space of the acceleration and deceleration phases stabilized at higher IDs (i.e., longer evoked MTs). With the distance covered during the acceleration phase decreasing with MT, this gave rise an increase in CVspace. During the deceleration phase relative spatial variability decreased at higher IDs, as the distance covered during this phase increased. In both tasks SDtime and CVtime of the acceleration and deceleration phases increased with MT (excepted for CVtime in Fitts deceleration phase), as was observed for the whole movement. As shown in Table 2, these observations were corroborated by ANOVAs with repeated measures on the factors ID (Fitts task) and Prescribed MT (Schmidt task).

#### Covariation of spatial and temporal variabilities

**Figure 2:**
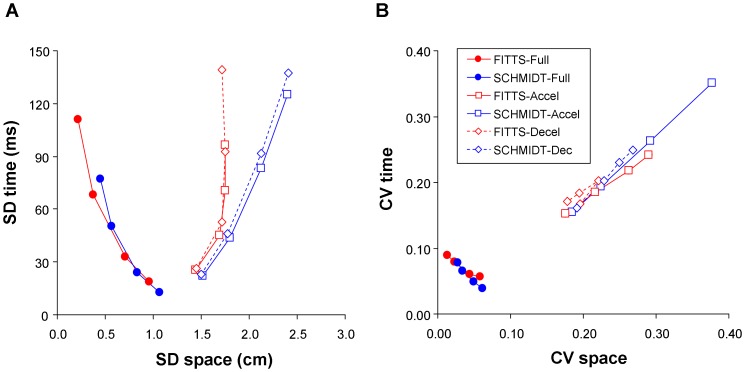
Relations between temporal and spatial variabilities observed in Experiment 2 (elastic force-field setting) over the full movement and the constituent acceleration and deceleration phases. Space-time relations for absolute variability (A) and relative variability (B).

**Figure 3:**
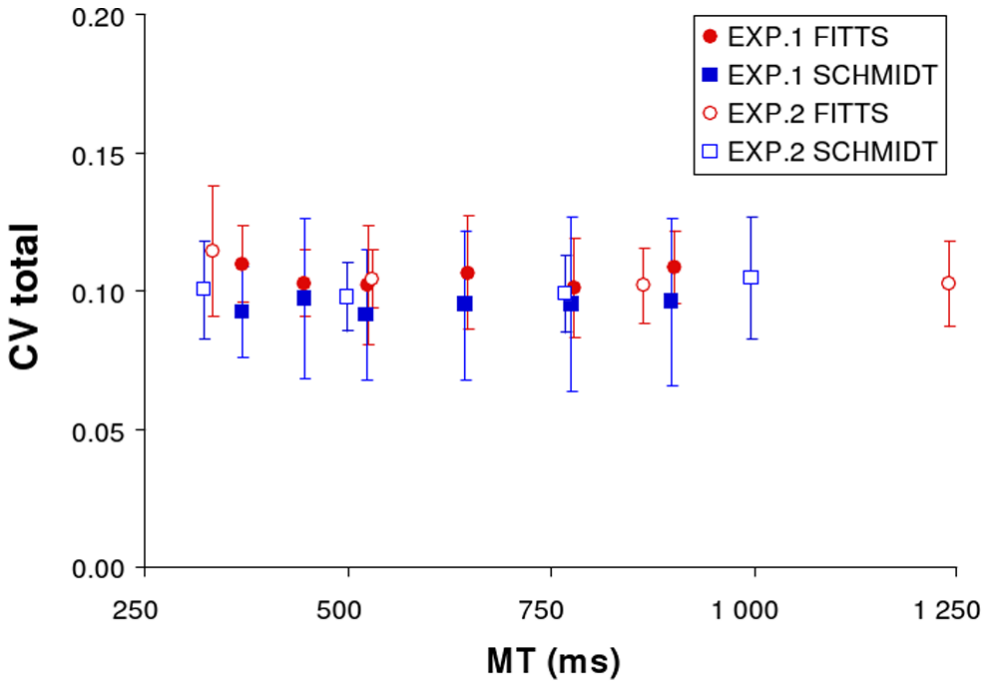
Total relative variability observed in Experiment 1 (standard setting) and Experiment 2 (elastic force-field setting). Total relative variability (CVtotal) is expressed as the sum of temporal (CVtime) and spatial (CVspace) variabilities. Each task (Fitts and Schmidt) is presented separately.

The results of Experiment 2 demonstrate the same phenomena observed in Experiment 1. For both the Fitts and Schmidt tasks, a trade-off between spatial and temporal variabilities was observed at the level of the full movement, whether variability be expressed in absolute (Fig. 2A) or relative (Fig. 2B) terms. SDspace and SDtime were again negatively correlated (Fitts: *r*(2)  = −0.96, *p*<0.05; Schmidt: *r*(2)  = −0.96, *p*<0.05). Similar results were obtained for correlations between CVspace and CVtime (Fitts: *r*(2)  = −0.98, *p*<0.05; Schmidt: *r*(2)  = −0.99, *p*<0.05). As in Experiment 1, CVtotal and MT were not systematically related, as revealed by correlation coefficients that remained far from significance for both tasks (*p*'s>0.2). One way ANOVAs with repeated measures on the factor ID (Fitts task) or Prescribed MT (Schmidt task) showed no significant effects (*F*(3, 27) <1.64 *p'*s>0.20). In the Fitts task the mean composite CV was 11.0±0.6% (SD across IDs), and in the Schmidt task it was 10.6±0.4% (SD across prescribed MTs). Figure 3 presents the stability of CVtotal across MTs in both tasks as well as in both experiments.

When movement phases were considered separately, spatial and temporal variabilities were found to covary positively no matter whether they were expressed in absolute (Fig. 2A) or in relative terms (Fig. 2B). Further examination of relative variability showed that for the acceleration phase the correlations between CVtime and CVspace were *r*(2) = +1.00, *p*<0.001 (Fitts task), and *r*(2)  = +1.00, *p*<0.001 (Schmidt task). For the deceleration phase the correlations between CVtime and CVspace were *r*(2)  = +0.83, *ns* (Fitts task), and *r*(2) = +1.00, *p*<0.001 (Schmidt task).

## Discussion

The goal of this study was to investigate how spatial and temporal variabilities of upper-limb reciprocal aiming movements were influenced by movement duration when the latter was manipulated directly (Schmidt task) or indirectly (Fitts task). With respect to this overall objective the following key findings were obtained. First, independent of how movement duration was manipulated (directly or indirectly), for a given movement amplitude both absolute and relative temporal variabilities increased as a function of movement duration, while both absolute and relative spatial variabilities decreased with movement duration. Second, manipulation of movement duration, whether direct or indirect, gave rise to a trade-off between spatial and temporal variabilities. Such trade-offs were observed whether we considered absolute or relative variabilities. Third, these findings held for the full movement but were not observed at the level of the individual acceleration and deceleration phases of movement. Separately, indeed, the acceleration and deceleration phases were characterized by greater relative spatial and temporal variabilities, as well as positive correlations between them over increases in movement duration. Finally, all the previous findings emerged whether the reciprocal aiming movements were performed under the standard setting or under the elastic force-field setting.

We first discuss the relations between movement duration and spatial and temporal variabilities separately, before turning our attention to the trade-off between them. We end the discussion by evoking different potential mechanisms.

### Relation between movement duration and spatial and temporal variabilities

As expected from the extensive body of work on the speed-accuracy trade-off in both experimental settings and in both reciprocal aiming tasks, smaller (relative and absolute) spatial variability was associated with longer MTs. Earlier work relying on discrete movement timing and interception tasks [22]–[27] also led us to expect the observed increase in absolute temporal variability with increasing movement duration. Still, we are not aware of earlier studies that have indeed reported this finding in the framework of amplitude-controlled reciprocal aiming tasks. Shafir and Brown [33] did find similar effects for single-joint movements, but not for multi-joint movements. We suggest that their hybrid aiming task, combining fixed target widths with metronome-prescribed movement durations may have obscured these effects at the level of hand movement. The fact that absolute temporal variability increased faster than linear, leading to an increase in relative temporal variability with increasing movement duration, is also a new finding in this framework.

### Trade-off between spatial and temporal variabilities

The main result of the current study was that spatial variability and temporal variability are strongly negatively correlated over variations in movement duration, when considering the whole movement. Indeed we found that both SDspace and CVspace decreased with MT while both SDtime and CVtime increased with MT. Although based on different experimental tasks and procedures, we expected that SDspace would decrease with MT, while SDtime would increase with MT. Thus, the trade-off between absolute spatial and temporal variabilities demonstrated in the present contribution is perhaps not surprising. It has however not been demonstrated before within a unique experimental paradigm. The trade-off between relative spatial and temporal variabilities was less expected and has, to our knowledge, not been identified earlier. We found moreover that CVtime and CVspace were related in such a way that their sum remained relatively constant (see Figure 3).

Harris and Wolpert ([6] but also see [3], [30], [45]–[47]) proposed that variability in motor performance arises (at least partly) from signal-dependent noise (SDN) in motor execution processes. Briefly, they assumed that neural commands carry signal-dependent noise whose standard deviation increases linearly with the absolute value of the neural control signal. As a result faster movements are characterized by noisier neural commands, which in turn diminishes movement accuracy. Although this scheme provides a theoretical account of the speed–accuracy trade-off described by Fitts' law [6], [47], it remains silent on the origins of absolute and relative temporal variability. In order to account for variability in MT within the framework of SDN, Van Beers and collaborators have proposed to introduce temporal noise in the neural commands [8], [9]. However, in that work the level of temporal noise was adjusted to match the observed variability in movement time: at present their approach therefore remains more descriptive than explanatory. We conclude that, at this stage, the SDN framework does not provide a satisfactory theoretical explanation of the trade-off between spatial and temporal variabilities demonstrated in the present contribution.

In order to get a better grasp on the control mechanisms underlying the emergence of spatial and temporal variabilities, we conducted separate analyses of spatial and temporal variabilities on the acceleration and deceleration phases of movement. Interestingly the results of these analyses contrasted substantially with those conducted on the full movement (see Figures 1 and 2). First, during the acceleration or deceleration phases of movement relative variability was typically higher than observed for the full movement. Second, during the acceleration and deceleration phases of movement, spatial and temporal variabilities (both absolute and relative) were positively correlated, while they were negatively correlated at the level of the full movement.

These observations indicate that the acceleration and deceleration phases were complementary rather than supplementary. In other words, rather than being independent of each other, with variability cumulating over the unfolding movement, the deceleration phase compensated for variability accumulated during the acceleration phase. Together with the differences observed between Fitts and Schmidt tasks this provides strong evidence for a structuring role of feedback mechanisms [37], [45].

In the introduction, the possibility that spatial and temporal variabilities could be positively correlated was envisaged based on the assumption that both types of variability could reflect similar corruptive processes in neural commands. Deep brain stimulation has indeed been demonstrated to improve both the spatial and the temporal accuracy of reciprocal aiming movements in multiple sclerosis patients [48]. However, the current study (also see [33]), does not support the view that a single mechanism is responsible for spatial and temporal variabilities in upper-limb reciprocal aiming movements. To account for the trade-off between relative spatial and temporal variabilities we propose two schemes. In the first one, this trade-off is viewed as a consequence of the limited availability of attentional resources. We reasoned that the minimization of spatial and temporal variabilities could be driven by separate processes that compete for attentional resources. In other words when spatial accuracy requirements are elevated (e.g., high ID), this would have detrimental effects on temporal accuracy, and vice versa. This scheme has attractive aspects because it not only accounts for opposite changes in variability with movement time, but also accounts for the fact that our values of CVtime are significantly higher than those typically reported in finger tapping experiments that did not carry spatial requirements (about 0.035 in [49]; 0.043 in [14]; 0.05 in [15]).

An alternative explanation is provided by the dynamic systems approach in which rhythmic movements are understood as self-sustained oscillators [50]–[52]. In this framework variability in movement is not simply taken to reflect noise, but as an indicator of the stability of the system. Hence, examining variability in motor behavior can be informative about stability properties of the neuromechanically instantiated oscillators producing that behavior. Mottet and Bootsma [52] demonstrated that the systematic changes in the kinematic patterns observed over different levels of difficulty during reciprocal aiming in a Fitts task could be adequately captured by variations in the parameters of an invariant dynamical structure, combining linear and cubic stiffness and linear and cubic damping terms. In the original paper presenting data from this contributions' Experiment 1 [38], we showed that this Rayleigh-Duffing (RD) model could also successfully account for the observed changes in the kinematic patterns observed during reciprocal aiming in a Schmidt task, thereby providing a unique theoretical framework for understanding both reciprocal aiming tasks. We suggest that the variations in the parameters of the RD model observed over task conditions [43], [52]–[60] and over tasks [38] affect not only the (average) kinematic patterns of movement, but, via the associated changes in stability characteristics, also the spatial and temporal variabilities. Interesting in this respect is that under conditions in which spatial variability was reduced we observed a larger contribution of cubic stiffness relative to linear stiffness; under conditions in which temporal variability was reduced we observed a larger contribution of cubic damping relative to linear damping. Although for the purpose of the current paper these relations can only be indicated qualitatively, there are thus suggestions in the data that the relation between spatial and temporal variabilities as revealed in the current paper could emerge from the stability characteristics of the dynamic regime that has to be setup to meet task demands.

### Robustness of the findings with respect to changes in dynamic environment

To test the robustness of the findings provided by the first experiment in which movement was performed by sliding a stylus over a graphic tablet, in the second experiment we reproduced our Schmidt and Fitts tasks in a situation in which movement was performed against an elastic load. Although changes in external force fields are known to impact kinematic and/or electromyographic variables [61]–[64], as well as the temporal structure of motor variability [65], all the main findings of Experiment 1 were replicated under the elastic force-field conditions of Experiment 2 (i.e. the increase in CVtime with MT, and the linear trade-off between temporal and spatial variabilities). These findings complement the study of Shafir and Brown [33] in which inertial loading of the forearm had no significant effect on relative spatial and temporal variabilities. Overall, it seems that the mechanisms underlying the trade-off between relative spatial and temporal variabilities are robust enough to accommodate not only changes in the experimental tasks, but also changes in the external force fields encountered.

Finally we would like also to acknowledge the robustness of Fitts' law that also persisted despite the adjunction of the elastic load. Although there is a previous account that Fitts' law holds under an elastic load [66], it is noteworthy that in that study the neutral elastic force position was located centrally between the targets, meaning that the assistance/hindrance of the elastic load was the same for back and forth movements. In contrast, in the current study the neutral position was positioned outside the range of movement, meaning that movements in one direction were assisted, while movements in the opposite direction were hindered. Overall, it appears that Fitts' Law is robust enough to account for the dynamics of reciprocal aiming movements despite this asymmetry in movement assistance. This conclusion fits well with other observations showing that Fitts' law also holds when artificially varying gravitational forces acting on the arm [67].

### Concluding comments

Based on our joint analysis of spatial and temporal properties of reciprocal aiming movements, the present study revealed the existence of a trade-off between spatial and temporal variabilities both at the level of SD (absolute variability) and CV (relative variability). Although this relationship may not hold over all types of movement it was robust enough to persist over variations in experimental protocols (evoked versus prescribed MT), and task settings (standard versus elastic force-field). Overall, although the reasons underlying this trade-off remain to be clarified, our results speak in favor of competitive processes minimizing spatial and temporal variabilities, even when the latter is not explicitly required by the task (i.e. Fitts task). Because greater variabilities and opposite trends were observed when analyzing separately the acceleration and deceleration phase of the movement, we suggest that the minimization of spatial and temporal variabilities within these phases is less relevant than over the whole movement.

## Supporting Information

Table S1 **Summary of duration and distance characteristics under the conditions of Experiment 1.** Means, standard deviations (SD) and coefficients of variation (CV) of Movement Time (MT) and Movement Distance (MD), for the full movement and for the acceleration and deceleration phases separately, in the (evoked MT) Fitts and (imposed MT) Schmidt task. ID =  Index of Difficulty. MTp =  prescribed movement time.
(DOC)

Table S2 **Summary of duration and distance characteristics under the conditions of Experiment 2.** Means, standard deviations (SD) and coefficients of variation (CV) of Movement Time (MT) and Movement distance (MD) for the full movement and for the acceleration and deceleration phases separately in the (evoked MT) Fitts and (imposed MT) Schmidt task. ID =  Index of Difficulty. MTp =  prescribed movement time.
(DOC)

## References

[1] pone.0097447-1. Woodworth RS (1899) Accuracy of voluntary movement. The Psychological Review: Monograph Supplements 3: i–114 10.1037/h0092992

[2] Fitts PM (1954) The information capacity of the human motor system in controlling the amplitude of movement. J Exp Psychol 47: 381–391. 13174710

[3] Schmidt RA, Zelaznik H, Hawkins B, Frank JS, Quinn JT Jr (1979) Motor-output variability: a theory for the accuracy of rapid motor acts. Psychol Rev 47: 415–451. 504536

[4] Meyer DE, Abrams RA, Kornblum S, Wright CE, Smith JE (1988) Optimality in human motor performance: ideal control of rapid aimed movements. Psychol Rev 95: 340–370. 3406245 10.1037/0033-295x.95.3.340

[5] Newell KM, Corcos DM (1993)Variability and Motor Control. Human Kinetics Publishers. 536 p.

[6] Harris CM, Wolpert DM (1998) Signal-dependent noise determines motor planning. Nature 394: 780–784 10.1038/29528 9723616

[7] Faisal AA, Selen LPJ, Wolpert DM (2008) Noise in the nervous system. Nat Rev Neurosci 9: 292–303 10.1038/nrn2258 18319728 PMC2631351

[8] Van Beers RJ, Haggard P, Wolpert DM (2004) The role of execution noise in movement variability. J Neurophysiol 91: 1050–1063 10.1152/jn.00652.2003 14561687

[9] Van Beers RJ (2007) The sources of variability in saccadic eye movements. J Neurosci 27: 8757–8770 10.1523/JNEUROSCI.2311-07.2007 17699658 PMC6672172

[10] Churchland MM, Afshar A, Shenoy KV (2006) A central source of movement variability. Neuron 52: 1085–1096 10.1016/j.neuron.2006.10.034 17178410 PMC1941679

[11] Gordon J, Ghilardi MF, Ghez C (1994) Accuracy of planar reaching movements. I. Independence of direction and extent variability. Exp Brain Res 99: 97–111. 7925800 10.1007/BF00241415

[12] Hancock PA, Newell KM (1985) The Movement Speed-Accuracy Relationship in Space-Time. In: Heuer PDH, Kleinbeck PDU, Schmidt D-PK-H, editors.Motor Behavior. Springer Berlin Heidelberg. pp.153–188. Available: http://link.springer.com/chapter/10.1007/978-3-642-69749-4_5 Accessed 2013 July 24.

[13] Wing AM, Kristofferson AB (1973) The timing of interresponse intervals. Perception and psychophysics 13: 455–460.

[14] Ivry RB, Hazeltine RE (1995) Perception and production of temporal intervals across a range of durations: evidence for a common timing mechanism. J Exp Psychol Hum Percept Perform 21: 3–18. 7707031 10.1037//0096-1523.21.1.3

[15] Sternad D, Dean WJ, Newell KM (2000) Force and timing variability in rhythmic unimanual tapping. J Mot Behav 32: 249–267. 10975273 10.1080/00222890009601376

[16] Lorås H, Sigmundsson H, Talcott JB, Öhberg F, Stensdotter AK (2012) Timing continuous or discontinuous movements across effectors specified by different pacing modalities and intervals. Exp Brain Res 220: 335–347 10.1007/s00221-012-3142-4 22710620

[17] Spencer RMC, Zelaznik HN (2003) Weber (slope) analyses of timing variability in tapping and drawing tasks. J Mot Behav 35: 371–381 10.1080/00222890309603157 14607774

[18] Bartlett NR, Bartlett SC (1959) Synchronization of a motor response with an anticipated sensory event. Psychol Rev 66: 203–218. 13668002 10.1037/h0046490

[19] Dalla Bella S, Palmer C (2011) Rate effects on timing, key velocity, and finger kinematics in piano performance. PLoS ONE 6: e20518 10.1371/journal.pone.0020518 21731615 PMC3121738

[20] Robertson SD, Zelaznik HN, Lantero DA, Bojczyk KG, Spencer RM, et al (1999) Correlations for timing consistency among tapping and drawing tasks: evidence against a single timing process for motor control. J Exp Psychol Hum Percept Perform 25: 1316–1330. 10531665 10.1037//0096-1523.25.5.1316

[21] Doumas M, Wing AM (2007) Timing and trajectory in rhythm production. J Exp Psychol Hum Percept Perform 33: 442–455 10.1037/0096-1523.33.2.442 17469978

[22] Newell KM, Hoshizaki L, Carlton MJ (1979) Movement time and velocity as determinants of movement timing accuracy. J Mot Behav 11: 49–58. 15186971 10.1080/00222895.1979.10735171

[23] Newell KM, Carlton LG, Carlton MJ (1980) Velocity as a factor in movement timing accuracy. J Mot Behav 12: 47–56. 15215067 10.1080/00222895.1980.10735204

[24] Newell KM, Carlton LG, Carlton MJ (1982) The relationship of impulse to response timing error. J Mot Behav 14: 24–45. 15151893 10.1080/00222895.1982.10735260

[25] Tresilian JR, Lonergan A (2002) Intercepting a moving target: effects of temporal precision constraints and movement amplitude. Exp Brain Res 142: 193–207 10.1007/s00221-001-0920-9 11807574

[26] Tresilian JR, Plooy A, Carroll TJ (2004) Constraints on the spatiotemporal accuracy of interceptive action: effects of target size on hitting a moving target. Exp Brain Res 155: 509–526 10.1007/s00221-003-1793-x 14999437

[27] Brouwer A-M, Smeets JBJ, Brenner E (2005) Hitting moving targets: effects of target speed and dimensions on movement time. Exp Brain Res 165: 28–36 10.1007/s00221-005-2277-y 15868174

[28] Tresilian JR, Plooy A (2006) Systematic changes in the duration and precision of interception in response to variation of amplitude and effector size. Exp Brain Res 171: 421–435 10.1007/s00221-005-0286-5 16307234

[29] Kim S, Carlton LG, Liu Y-T, Newell KM (1999) Impulse and Movement Space-Time Variability. J Mot Behav 31: 341–357 10.1080/00222899909600999 11177642

[30] Zelaznik HN, Mone S, McCabe GP, Thaman C (1988) Role of temporal and spatial precision in determining the nature of the speed-accuracy trade-off in aimed-hand movements. Journal of Experimental Psychology: Human Perception and Performance 14: 221–230 10.1037/0096-1523.14.2.221

[31] Fitts PM, Peterson JR (1964) Information capacity of discrete motor responses. J Exp Psychol 67: 103–112. 14114905 10.1037/h0045689

[32] Plamondon R, Alimi AM (1997) Speed/accuracy trade-offs in target-directed movements. Behav Brain Sci 20: 279–303 discussion 303-349. 10096999 10.1017/s0140525x97001441

[33] Shafir T, Brown SH (2010) Timing and the control of rhythmic upper-limb movements. J Mot Behav 42: 71–84 10.1080/00222890903397137 20051350

[34] Danion F, Varraine E, Bonnard M, Pailhous J (2003) Stride variability in human gait: the effect of stride frequency and stride length. Gait Posture 18: 69–77. 12855302 10.1016/s0966-6362(03)00030-4

[35] Danion F, Duarte M, Grosjean M (2006) Variability of reciprocal aiming movements during standing: the effect of amplitude and frequency. Gait Posture 23: 173–179 10.1016/j.gaitpost.2005.01.005 16399513

[36] Elliott D, Helsen WF, Chua R (2001) A century later: Woodworth's (1899) two-component model of goal-directed aiming. Psychol Bull 127: 342–357. 11393300 10.1037/0033-2909.127.3.342

[37] Elliott D, Hansen S, Grierson LEM, Lyons J, Bennett SJ, et al (2010) Goal-directed aiming: two components but multiple processes. Psychol Bull 136: 1023–1044 10.1037/a0020958 20822209

[38] Bongers RM, Fernandez L, Bootsma RJ (2009) Linear and logarithmic speed-accuracy trade-offs in reciprocal aiming result from task-specific parameterization of an invariant underlying dynamics. J Exp Psychol Hum Percept Perform 35: 1443–1457 10.1037/a0015783 19803648

[39] Danion F, Sarlegna FR (2007) Can the human brain predict the consequences of arm movement corrections when transporting an object? Hints from grip force adjustments. J Neurosci 27: 12839–12843 10.1523/JNEUROSCI.3110-07.2007 18032655 PMC6673284

[40] Danion F, Jirsa VK (2010) Motor prediction at the edge of instability: alteration of grip force control during changes in bimanual coordination. J Exp Psychol Hum Percept Perform 36: 1684–1692 10.1037/a0020672 21038992

[41] Sarlegna FR, Baud-Bovy G, Danion F (2010) Delayed visual feedback affects both manual tracking and grip force control when transporting a handheld object. J Neurophysiol 104: 641–653 10.1152/jn.00174.2010 20538774

[42] MacKenzie KM, Kerr SR, Rouse MW, DeLand PN (1987) Study of accommodative facility testing reliability. American Journal of Optometry and Physiological Optics 64: 186–194. 3578484 10.1097/00006324-198703000-00005

[43] Bootsma RJ, Fernandez L, Mottet D (2004) Behind Fitts' law: kinematic patterns in goal-directed movements. International Journal of Human-Computer Studies 61: 811–821 10.1016/j.ijhcs.2004.09.004

[44] Huys R, Fernandez L, Bootsma RJ, Jirsa VK (2010) Fitts' law is not continuous in reciprocal aiming. Proc Biol Sci 277: 1179–1184 10.1098/rspb.2009.1954 20018791 PMC2842815

[45] Todorov E, Jordan MI (2002) Optimal feedback control as a theory of motor coordination. Nat Neurosci 5: 1226–1235 10.1038/nn963 12404008

[46] Van Beers RJ, Baraduc P, Wolpert DM (2002) Role of uncertainty in sensorimotor control. Philos Trans R Soc Lond, B, Biol Sci 357: 1137–1145 10.1098/rstb.2002.1101 12217180 PMC1693018

[47] Guigon E, Baraduc P, Desmurget M (2008) Computational motor control: feedback and accuracy. Eur J Neurosci 27: 1003–1016 10.1111/j.1460-9568.2008.06028.x 18279368

[48] Spiegel J, Dillmann U, Moringlane JR (2007) Quantification of temporal and spatial accuracy of alternating arm movements in multiple sclerosis patients treated with deep brain stimulation of the thalamic ventralis intermedius nucleus (VIM). Zentralbl Neurochir 68: 67–72 10.1055/s-2007-977739 17614086

[49] Semjen A, Schulze HH, Vorberg D (2000) Timing precision in continuation and synchronization tapping. Psychol Res 63: 137–147. 10946587 10.1007/pl00008172

[50] Haken H, Kelso JA, Bunz H (1985) A theoretical model of phase transitions in human hand movements. Biol Cybern 51: 347–356. 3978150 10.1007/BF00336922

[51] Beek PJ, Schmidt RC, Morris AW, Sim MY, Turvey MT (1995) Linear and nonlinear stiffness and friction in biological rhythmic movements. Biol Cybern 73: 499–507. 8527496 10.1007/BF00199542

[52] Mottet D, Bootsma RJ (1999) The dynamics of goal-directed rhythmical aiming. Biol Cybern 80: 235–245. 10326240 10.1007/s004220050521

[53] Billon M, Bootsma RJ, Mottet D (2000) The dynamics of human isometric pointing movements under varying accuracy requirements. Neurosci Lett 286: 49–52. 10822150 10.1016/s0304-3940(00)01089-2

[54] Mottet D, Bootsma RJ (2001) The dynamics of rhythmical aiming in 2D task space: relation between geometry and kinematics under examination. Hum Mov Sci 20: 213v241. 11517670 10.1016/s0167-9457(01)00038-0

[55] Mottet D, Guiard Y, Ferrand T, Bootsma RJ (2001) Two-handed performance of a rhythmical fitts task by individuals and dyads. J Exp Psychol Hum Percept Perform 27: 1275–1286. 11766924 10.1037//0096-1523.27.6.1275

[56] Bootsma RJ, Boulard M, Fernandez L, Mottet D (2002) Informational constraints in human precision aiming. Neurosci Lett 333: 141–145. 12419500 10.1016/s0304-3940(02)01003-0

[57] Bootsma RJ, Mottet D (2004) Dynamic Invariance in Goal-Directed Aiming. Ecological Psychology 16: 55–60 10.1207/s15326969eco16017

[58] Fernandez L, Bootsma RJ (2004) Effects of biomechanical and task constraints on the organization of movement in precision aiming. Exp Brain Res 159: 458–466 10.1007/s00221-004-1964-4 15252700

[59] Fernandez L, Warren WH, Bootsma RJ (2006) Kinematic adaptation to sudden changes in visual task constraints during reciprocal aiming. Hum Mov Sci 25: 695–717 10.1016/j.humov.2006.05.001 16859793

[60] Fernandez L, Bootsma RJ (2008) Non-linear gaining in precision aiming: making Fitts' task a bit easier. Acta Psychol (Amst) 129: 217–227 10.1016/j.actpsy.2008.06.001 18632086

[61] Bonnard M, Pailhous J, Danion F (1997) Intentional On-Line Adaptation of Rhythmic Movements During a Hyper- to Microgravity Change. Motor Control 1: 247–262.

[62] Flanagan JR, Wing AM (1997) The role of internal models in motion planning and control: evidence from grip force adjustments during movements of hand-held loads. Journal of Neuroscience 17: 1519–1528. 9006993 10.1523/JNEUROSCI.17-04-01519.1997 PMC6793733

[63] Thoroughman KA, Shadmehr R (1999) Electromyographic Correlates of Learning an Internal Model of Reaching Movements. J Neurosci 19: 8573–8588. 10493757 10.1523/JNEUROSCI.19-19-08573.1999 PMC6783008

[64] Mackey DC, Meichenbaum DP, Shemmell J, Riek S, Carson RG (2002) Neural compensation for compliant loads during rhythmic movement. Exp Brain Res 142: 409–417 10.1007/s00221-001-0946-z 11819050

[65] Wu HG, Miyamoto YR, Castro LNG, Olveczky BP, Smith MA (2014) Temporal structure of motor variability is dynamically regulated and predicts motor learning ability. Nat Neurosci 17: 312–321 10.1038/nn.3616 24413700 PMC4442489

[66] Casiez G, Vogel D (2008) The effect of spring stiffness and control gain with an elastic rate control pointing device. Proceedings of the twenty-sixth annual SIGCHI conference on Human factors in computing systems. CHI '08. New York, NY, USA: ACM. pp. 1709–1718. Available: http://doi.acm.org/10.1145/1357054.1357321. Accessed 2012 May 30.

[67] Ciofani G, Migliore A, Mazzei D, Carrozza MC, Dario P (2010) Modification of Pointing Performance in Altered Gravitational Environments. Microgravity Sci Technol 22: 123–128 10.1007/s12217-009-9163-3

