# Supplementary material for: The Trade-Off between Spatial and Temporal Variabilities in Reciprocal Upper-Limb Aiming Movements of Different Durations
Source: PLoS One. 2014 May 16;9(5):e97447. doi: 10.1371/journal.pone.0097447 (PMC4023964; doi:10.1371/journal.pone.0097447)
Supplement: Table S2 — Summary of duration and distance characteristics under the conditions of Experiment 2. Means, standard deviations (SD) and coefficients of variation (CV) of Movement Time (MT) and Movement distance (MD) for the full movement and for the acceleration and deceleration phases separately in the (evoked MT) Fitts and (imposed MT) Schmidt task. ID = Index of Difficulty. MTp = prescribed movement time. (DOC) [file pone.0097447.s002.doc]

**Table S2: Summary of duration and distance characteristics under the conditions of Experiment 2.** Means, standard deviations (SD) and coefficients of variation (CV) of Movement Time (MT) and Movement distance (MD) for the full movement and for the acceleration and deceleration phases separately in the (evoked MT) Fitts and (imposed MT) Schmidt task. ID = Index of Difficulty. MTp = prescribed movement time.

| **Fitts Task** | | | | | | | |
| --- | --- | --- | --- | --- | --- | --- | --- |
|  |  | *Total Movement* | | *Acceleration Phase* | | *Deceleration Phase* | |
| **ID** |  | MT (ms) | MD (cm) | MT (ms) | MD (cm) | MT (ms) | MD (cm) |
| **3** | Mean | 334.3 | 16.46 | 174.0 | 8.66 | 160.3 | 7.80 |
|  | SD | 19.0 | 0.96 | 25.3 | 1.44 | 26.5 | 1.45 |
|  | CV | 0.056 | 0.058 | 0.152 | 0.176 | 0.167 | 0.195 |
| **4** | Mean | 530.9 | 16.27 | 256.8 | 8.13 | 274.1 | 8.14 |
|  | SD | 32.9 | 0.71 | 45.3 | 1.69 | 52.6 | 1.72 |
|  | CV | 0.061 | 0.044 | 0.185 | 0.217 | 0.202 | 0.220 |
| **5** | Mean | 864.0 | 16.18 | 332.3 | 6.75 | 531.7 | 9.43 |
|  | SD | 68.4 | 0.37 | 70.5 | 1.75 | 92.9 | 1.75 |
|  | CV | 0.079 | 0.023 | 0.218 | 0.262 | 0.184 | 0.194 |
| **6** | Mean | 1240.5 | 16.21 | 402.9 | 6.15 | 837.6 | 10.06 |
|  | SD | 110.9 | 0.21 | 96.6 | 1.75 | 139.3 | 1.72 |
|  | CV | 0.090 | 0.013 | 0.242 | 0.290 | 10.058 | 0.178 |
|  |  |  |  |  |  |  |  |
| **Schmidt Task** | | | | | | | |
|  |  | *Total Movement* | | *Acceleration Phase* | | *Deceleration Phase* | |
| **MTp (ms)** |  | MT (ms) | MD (cm) | MT (ms) | MD (cm) | MT (ms) | MD (cm) |
| **322.6** | Mean | 321.9 | 17.28 | 168.6 | 9.09 | 153.3 | 8.20 |
|  | SD | 12.6 | 1.07 | 21.9 | 1.52 | 22.9 | 1.50 |
|  | CV | 0.039 | 0.061 | 0.154 | 0.184 | 0.161 | 0.191 |
| **500.0** | Mean | 499.0 | 16.79 | 246.9 | 8.50 | 252.1 | 8.30 |
|  | SD | 24.2 | 0.83 | 43.9 | 1.80 | 46.2 | 1.78 |
|  | CV | 0.049 | 0.049 | 0.194 | 0.224 | 0.203 | 0.228 |
| **769.2** | Mean | 768.1 | 16.66 | 332.0 | 7.54 | 436.0 | 9.12 |
|  | SD | 50.1 | 0.56 | 83.2 | 2.13 | 91.5 | 2.12 |
|  | CV | 0.065 | 0.034 | 0.262 | 0.293 | 0.231 | 0.250 |
| **1000.0** | Mean | 997.2 | 16.59 | 385.7 | 6.83 | 611.5 | 9.76 |
|  | SD | 77.2 | 0.45 | 124.9 | 2.40 | 137.4 | 2.41 |
|  | CV | 0.077 | 0.027 | 0.351 | 0.377 | 0.249 | 0.269 |
